# Chemopreventive Activities of Sulforaphane and Its Metabolites in Human Hepatoma HepG2 Cells

**DOI:** 10.3390/nu10050585

**Published:** 2018-05-09

**Authors:** Peng Liu, Wei Wang, Zhigang Zhou, Andrew J. O. Smith, Richard P. Bowater, Ian Michael Wormstone, Yuqiong Chen, Yongping Bao

**Affiliations:** 1 Norwich Medical School, University of East Anglia, Norfolk, Norwich NR4 7UQ, UK; P.Liu5@uea.ac.uk (P.L.); wei.wang@uea.ac.uk (W.W.); zhigang.zhou@ipswichhospital.nhs.uk (Z.Z.); 2 School of Biological Sciences, University of East Anglia, Norwich Research Park, Norwich, Norfolk NR4 7TJ, UK; a.j.smith3@uea.ac.uk (A.J.O.S.); r.bowater@uea.ac.uk (R.P.B.); I.M.Wormstone@uea.ac.uk (I.M.W.); 3 College of Horticulture and Forestry Science Huazhong Agricultural University, Wuhan 430070, China; chenyq@mail.hzau.edu.cn

**Keywords:** sulforaphane, chemoprevention, sulforaphane metabolites, Nrf2, GSH

## Abstract

Sulforaphane (SFN) exhibits chemopreventive effects through various mechanisms. However, few studies have focused on the bioactivities of its metabolites. Here, three metabolites derived from SFN were studied, known as sulforaphane glutathione, sulforaphane cysteine and sulforaphane-*N*-acetylcysteine. Their effects on cell viability, DNA damage, tumorigenicity, cell migration and adhesion were measured in human hepatoma HepG2 cells, and their anti-angiogenetic effects were determined in a 3D co-culture model of human umbilical vein endothelial cells (HUVECs) and pericytes. Results indicated that these metabolites at high doses decreased cancer cell viability, induced DNA damage and inhibited motility, and impaired endothelial cell migration and tube formation. Additionally, pre-treatment with low doses of SFN metabolites protected against H_2_O_2_ challenge. The activation of the nuclear factor E2-related factor 2 (Nrf2)-antioxidant response element (ARE) pathway and the induction of intracellular glutathione (GSH) played an important role in the cytoprotective effects of SFN metabolites. In conclusion, SFN metabolites exhibited similar cytotoxic and cytoprotective effects to SFN, which proves the necessity to study the mechanisms of action of not only SFN but also of its metabolites. Based on the different tissue distribution profiles of these metabolites, the most relevant chemical forms can be selected for targeted chemoprevention.

## 1. Introduction

The concept of chemoprevention is defined as the use of non-toxic chemicals to prevent or interfere with the development or progression of the neoplastic process that leads to cancer [1]. Chemoprevention by dietary phytochemicals is believed to be an ideal strategy for cancer management due to its readily applicable, acceptable and accessible nature [2,3,4]. Sulforaphane (1-isothiocyanate-(4-(methylsulfinyl) butane, SFN), first isolated from broccoli in 1992 [5] as an inducer of phase II detoxification enzymes, has been the subject of intensive research interest due to its chemopreventive effects [6].

**Figure 1:**
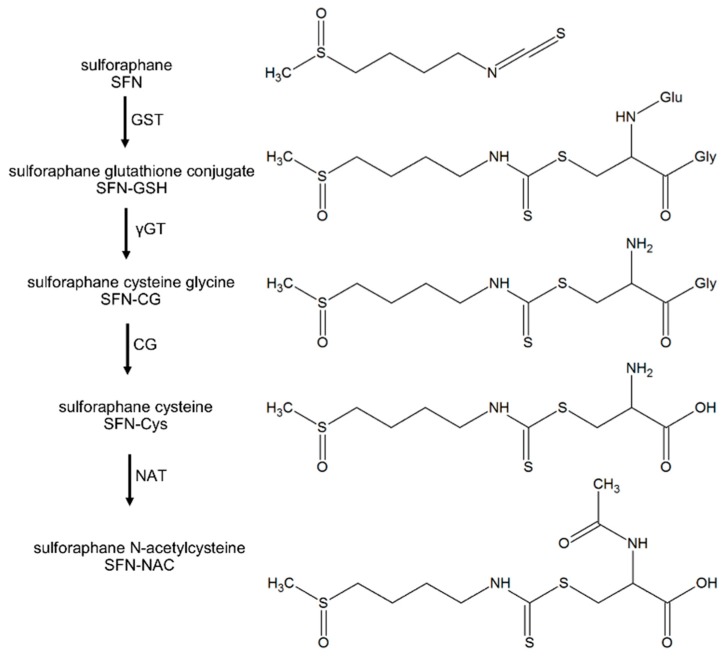
The mercapturic acid pathway of sulforaphane (SFN). GSTs, glutathione S-transferases; γ-GT, γ-glutamyl transferase; CG, cysteinylglycinase; NAT, *N*-acetyl transferase. Structures adopted from [7] and created using ACD/ChemSketch (Version C40H41, Advanced Chemistry Development, Inc., Toronto, ON, Canada).

SFN undergoes extensive metabolism via the mercapturic acid pathway. It first binds with glutathione (GSH) to form the corresponding conjugate (SFN-GSH), which then undergoes further enzymatic cleavage sequentially to the cysteinylglycine conjugate, cysteine conjugate (SFN-Cys), and *N*-acetylcysteine conjugates (SFN-NAC) (Figure 1) [7]. It has not been determined whether the bioactivity of SFN is due to these conjugates or to the parent SFN released by deconjugation reactions. According to Conaway et al., the half-life of decomposition for the Cys conjugates were several fold shorter than that of respective GSH conjugates, while NAC conjugates had the longest, at pH 7.4 and 37 °C [8]. Other studies agreed with Conaway et al. that the stability of SFN metabolites increased as pH decreased; and that SFN-NAC showed the longest decomposition half-life amongst all the metabolites found in human plasma [9]. It was speculated that the conjugates could be regarded as a transport form of SFN as they are unstable and readily dissociate to SFN or undergo exchange reactions with free thiols [10]. However, the greater solubility in aqueous media of these metabolites plus the different distributions of concentrations observed in the process of metabolism, suggests that they would be a preferred form for clinical chemoprevention trials in certain cases. For example, SFN and SFN-GSH concentrations in the small intestine were 3–13 nmol/g of tissue and 14–32 nmol/g of tissue respectively in a mouse feeding model [11]. SFN-Cys and SFN-NAC showed longer half-lives in circulation compared to SFN [12]. Especially SFN-NAC, the major SFN excretory product found in urine and at much higher concentrations than in plasma [13], has been studied to target bladder cancer [14].

Several studies have identified these metabolites as potent chemopreventive agents. Both SFN and SFN-GSH can significantly increase the mRNA levels of UGT1A1 and GSTA1, which are major isoforms of the human UDP-Glucuronosyltransferases (UGTs, EC 2.4.1.17) and glutathione transferases (GSTs, EC 2.5.1.18), in HepG2 and HT29 cells. Their functional induction of glucuronidation may contribute to the detoxification of carcinogens [15]. SFN-Cys was reported to induce apoptosis in human non-small lung cancer cells [16] and to suppress invasion in human prostate cancer cells [17]. SFN-NAC has been shown to exhibit potent chemopreventive activities that are equal to or greater than SFN. In a human prostate cancer LNCaP cell line model they showed similar potential to induce growth arrest and apoptosis [18] and they also inhibited lung adenoma induced by tobacco carcinogens and the development of adenomas to adenocarcinomas in mice [19]. In murine hepatoma cells, both SFN and SFN-NAC caused dose-related cell growth inhibition and NAD(P)H quinone dehydrogenase 1 (NQO1) induction [20]. The activity and expression of up-regulated histone deacetylases (HDACs) may be associated with the epigenetic repression of the tumor suppressor genes and the dysregulation of cell differentiation, proliferation, apoptosis, invasion and metastasis [21]. Dashwood et al. have shown that SFN, SFN-Cys and SFN-NAC act as HDAC inhibitors [22], which represents an important part of their chemopreventive activities. Conversely, SFN is known to inhibit the activities of cytochromes P450 (CYPs), namely CYP3A4 and CYP2D6, while SFN-GSH, SFN-Cys and SFN-NAC only showed inhibition of CYP2D6 in human liver microsomes [23]. This indicates there could be possible differential effects of SFN and its metabolites on the inhibition of carcinogen bioactivation. Others have reported that while SFN exhibited a protective effect against azoxymethane induced colonic aberrant crypt foci in F344 rats feeding, SFN-NAC did not [24]. Moreover, the induction of antioxidant response element (ARE) by SFN was found to be 8.6-fold higher than that of SFN-NAC in HepG2-C8 cells [25].

Since there are very few studies that have focused on the bioactivity of SFN metabolites, this study aimed to compare the cytotoxic and cytoprotective effect of SFN with that of its metabolites, SFN-GSH, SFN-Cys, and SFN-NAC, thereby shedding new light on their possible roles in chemoprevention.

## 2. Experimental Methods

### 2.1. Reagents

SFN and its metabolites were purchased from Toronto Research Chemicals (Toronto, ON, Canada). Complete protease inhibitors were obtained from Roche Applied Science (West Sussex, UK). Primary antibodies to nuclear factor E2-related factor 2 (Nrf2), thioredoxin reductase 1 (TrxR1), NQO1, SRC associated in mitosis of 68 kDa (SAM), β-actin, and the HRP-conjugated secondary antibodies were all purchased from Santa Cruz Biotechnology (Heidelberg, Germany). Anti-human CD31 was purchased from BD Biosciences (Oxford, UK). The Cy3 conjugated secondary antibodies were purchased from Jackson Immuno Research (West Grove, PA, USA). Nrf2 siRNA was obtained from Applied Biosystems (Warrington, UK). (Sense strand: 5′-CCUUAUAUCUCGAAGUUUUtt-3′; antisense strand: 5′-AAAACUUCGAGAUAAGGtg-3′). AllStars siRNA and HiPerFect transfection reagent were purchased from Qiagen (Manchester, UK) Unless otherwise indicated, all reagents were purchased from Sigma-Aldrich (Dorset, UK).

The level of SFN and its metabolites used in in vitro studies (0–10 µM) are within the range that could reasonably be expected to be encountered in vivo [26]. Higher doses were used to investigate their toxic effects. The molecular forms of these nutrients used in in vitro studies are the same as those which the cell type in the test would encounter in vivo.

### 2.2. Cell Culture

Human umbilical vein endothelial cells (HUVECs) were obtained from TCS Cellworks (Buckingham, UK) and murine MII perivascular cells (M2) were isolated as previously described [27]. For all experiments, HUVECs were used between passages 5 and 9, M2 was used between passages 35 and 40. Both cell lines were grown in flasks coated with 10 µg/mL type-I collagen. HUVECs were cultured in Endothelial Cell Growth Medium 2 (PromoCell, Heidelberg, Germany) supplemented with antibiotics (penicillin 100 U/mL and streptomycin 100 µg/mL) at 37 °C, 5% (*v*/*v*) CO_2_. M2 and human hepatoma cells HepG2 cells were cultured in Dulbecco’s modified Eagle’s medium (DMEM) supplemented with 10% foetal bovine serum (FBS), 2 mM glutamine, and antibiotics as above.

### 2.3. Cell Viability Assay

The 3-(4,5-dimethylthiazol-2-yl)-2,5 diphenyl tetrazolium bromide (MTT) assay was used to determine the toxicity as well as the protective effect of SFN and its metabolites towards cultured cells. Cells were seeded in 96-well plates and allowed to grow to 70–80% confluence. For the toxicity study, cells were treated with different doses of SFN or its metabolites for 24 h with dimethyl sulfoxide (DMSO) (0.1%) as control. For the protective study, cells were pre-treated with 10 µM SFN or metabolites for 24 h and then incubated with 700 µM H_2_O_2_ for another 24 h. After discarding the medium, 100 μL MTT (5 mg/mL) was added and incubated at 37 °C for 1 h. The formazan formed was then dissolved in 100 μL DMSO per well. The absorbance was determined using a microplate reader (BMG Labtech Ltd., Bucks, UK) at 550 nm with a 650 nm reference.

### 2.4. Alkaline Comet Assay

The alkaline comet assay, or single-cell gel electrophoresis, was used to assess levels of DNA strand breaks in individual cells [28]. HepG2 cells were seeded in 24-well plates and allowed to grow to 70–80% confluence. Then cells were treated with 20 µM SFN or metabolites for 24 h with DMSO (0.1%) as control. Otherwise, cells were pre-treated with 5 µM SFN or metabolites for 24 h, followed by 60 µM H_2_O_2_ for 30 min. Cells were harvested and subjected to an alkaline comet assay as described previously [29]. For each sample, at least 100 comets were randomly analyzed using Comet Assay IV Lite analysis software (Perceptive Instruments, Bury St Edmunds, UK). Levels of DNA strand breaks were expressed as tail intensity (% DNA in the comet tail) for statistical analysis.

### 2.5. Colony Formation Assay

HepG2 cells were seeded in 6-well plates at 2 × 10^5^ cell/mL and incubated for 24 h. Then cells were treated with 1.25, 5 and 20 µM SFN; or 10 µM SFN or metabolites for another 24 h. In both conditions, DMSO (0.1%) was used as control. After that, cells were trypsinized to make single-cell suspensions and seeded in new 6-well plates at 2000 cell/well (in triplicate) for each treatment group. Cells were maintained for at least 14 days to form colonies. The media was replaced every 3 days. Colonies were then fixed with ice-cold methanol and stained with 0.1% crystal violet for 30 min. For quantitative analysis, 1 mL 33% acetic acid was added to each well, then the plates were shaken for 1 h. The absorbance at 560 nm of each well was measured in the microplate reader (BMG Labtech Ltd., Aylesbury, UK). Colony formation % = (A560 test/A560 control) × 100%. Results were given as means and standard deviations of three independent experiments with triplicate samples for every treatment condition.

### 2.6. Wound Assay

The migration rates of HepG2 or HUVEC cells were assessed by the wound assay. Cells were seeded in 24-well plates at 2 × 10^5^ cells/mL and allowed to grow to 100% confluence. Wound gaps were made with a 1 mL pipette tip horizontally across the center of the wells. After debris were removed by gently washing with medium, the wells were filled with fresh medium containing different treatments and vehicle control. Each treatment was performed at least in triplicate. Cells were incubated for a further 48 or 12 h for HepG2 or HUVECs respectively, then stained with 1% crystal violet for imaging on an inverted microscope at 5× magnification. The wound area was quantitatively evaluated using ImageJ [30], and at least 10 pictures were used in each treatment.

### 2.7. Cell Adhesion Assay

Plates with 96-wells were coated with 50 μL of 10 µg/mL fibronectin (R&D Systems, Minneapolis, MN, USA) or rat tail collagen I (Merck KGaA, Darmstadt, Germany) or poly-l-lysine (PLL) overnight at 4 °C, and then blocked with 1% bovine serum albumin (BSA, Thermo Scientific, Waltham, MA USA) for 1 h at 20 °C. HepG2 cells were seeded in the coated plates at 5 × 10^4^ cells/well in serum-free medium with treatment (12 replicates for each treatment), and incubated under normal growth conditions for 1.5 h. Unattached cells were removed by three PBS washes. Then 50 μL of 4% paraformaldehyde (PFA) was added to each well for 10 min at 20 °C to fix the adherent cells. Plates were washed with PBS again and stained immediately with methylene blue for 30 min. The wells were then washed with dH_2_O to remove excess stain. To quantify the adherent cells, 100 μL de-staining buffer (50% ethanol in 0.1 M HCl) was added to each well for 10 min, and the absorbance was measured at 630 nm. Cell adhesion (%) was determined as (A630nm (test))/(A630nm (control)) × 100%. Three independent assays were conducted per experimental design.

### 2.8. Tube Formation in a 3D Model

HUVEC and M2 were co-cultured in collagen type I gel as described previously [31]. Medium containing 10 µM SFN or its metabolites with DMSO (0.1%) as control were added to the top of the collagen gel, and were changed every 48 h. At day 5, 3D collagen cultures were fixed, immunostained with CD31 and counterstained with DAPI. At least five random fields from each sample were imaged by fluorescence microscopy (Axioplan2, Carl Zeiss). For quantitative measurement, the total lengths of CD31-positive tube-like structures per area (mm/mm^2^) were analyzed by Volocity 4.0 (Improvision, Coventry, UK).

### 2.9. Western Blotting

For total protein extraction, cells were seeded in 6-well plates and allowed to grow to 70–80% confluence. Then cells were treated with 10 µM SFN or its metabolites for 24 h with DMSO (0.1%) as control. For the nuclear protein extraction, cells were seeded in 10 cm dishes and allowed to grow to 70–80% confluence. Then cells were treated with 10 µM SFN or its metabolites for 4 or 18 h with DMSO (0.1%) as control. The extraction was performed as described previously [32].

Protein concentration was determined by the Bradford assay to ensure equal levels of samples were loaded onto 10% SDS-polyacrylamide gels. After electrophoresis, proteins were transferred to polyvinylidene difluoride (PVDF) membrane, which was then blocked with 5% fat free milk in PBST (PBS with 0.01% Tween 20) for 1 h, incubated with primary antibody overnight at 4 °C and HRP-conjugated secondary antibody for 1 h. Immunoreactivity was determined by a chemiluminescence detection kit (GE Healthcare, Amersham, UK) and quantified by ImageJ [30].

### 2.10. Knockdown Nrf2 by siRNA

Reverse transfection of adherent cells in 96-well plates was performed according to the manufacturer’s protocol. siRNA (12.5 ng) was spotted in 25 μL of RNase-free water into each well (40 nM) of a 96-well plate. A mix of 0.75 μL of HiPerFect transfection reagent and 24.25 μL of culture medium without serum or antibiotics was then added to each well. The plate was incubated for 5–10 min at 20 °C to allow the formation of transfection complexes. HepG2 cells were then seeded at a density of 1 × 10^4^ cells/well in 150 μL of culture medium on top of the transfection complex. In each well, the final siRNA concentration was 5 nM. Cells were cultured under normal growth conditions for an additional 24 h. The MTT assay was then performed as described.

### 2.11. HPLC Analysis of Intracellular GSH

Approximately 1 × 10^6^ HepG2 cells were collected after 24 h treatment of 10 µM SFN or its metabolites with DMSO (0.1%) as control. Cells were washed twice in PBS and suspended in 1:4 volume of 5 mM diethylenetriaminepentaacetic acid and 50 mM methanesulfonic acid. After three freeze-thaw cycles and centrifugation at 12,000× *g* for 10 min, the GSH-containing supernatants were obtained and kept on ice for protein quantification. In order to obtain a true measurement of the amount of reduced GSH in living cells, GSH was derivatized using monobrow mobimane (mBBr) [33], a weakly fluorescent reagent that can freely cross the cell membrane. The procedure was performed as described previously [34]. The GSH-mBBr adduct was then measured by high-performance liquid chromatography (HPLC) with fluorescence detection. The separation was performed on a HiChrom ACE-AR C18 reversed phase column (4.6 × 250 mm, 5 μm, Phenomenex) with Solvent A (0.25%, *v*/*v* acetic acid and 10% methanol, pH 4). Samples were eluted with a gradient of Solvent B (90% methanol) at 1.0 mL/min flow rate as follows: 0–10 min 0% Solvent B; 10–11 min 50% Solvent B; 11–15 min 100% Solvent B; 16–20 min 0% Solvent B. Detection was conducted with excitation at 385 nm and emission at 460 nm. The gain of GSH-mBBr adduct eluted at 8.9 min and was quantified from a standard curve. The level of GSH was expressed as nmol/mg of cellular soluble protein.

### 2.12. Statistics

Data were represented as the mean ± SD (standard deviation). A Student’s *t* test analysis was performed to determine any statistical difference between two groups. One-way ANOVA with Tukey’s post hoc analysis was used to assess multiple groups when all or many pairwise comparisons were of interest. A *p* value < 0.05 was considered statistically significant.

## 3. Results

### 3.1. Cytotoxicity, Genotoxicity and Tumorigenicity of SFN vs. Its Metabolites

**Figure 2:**
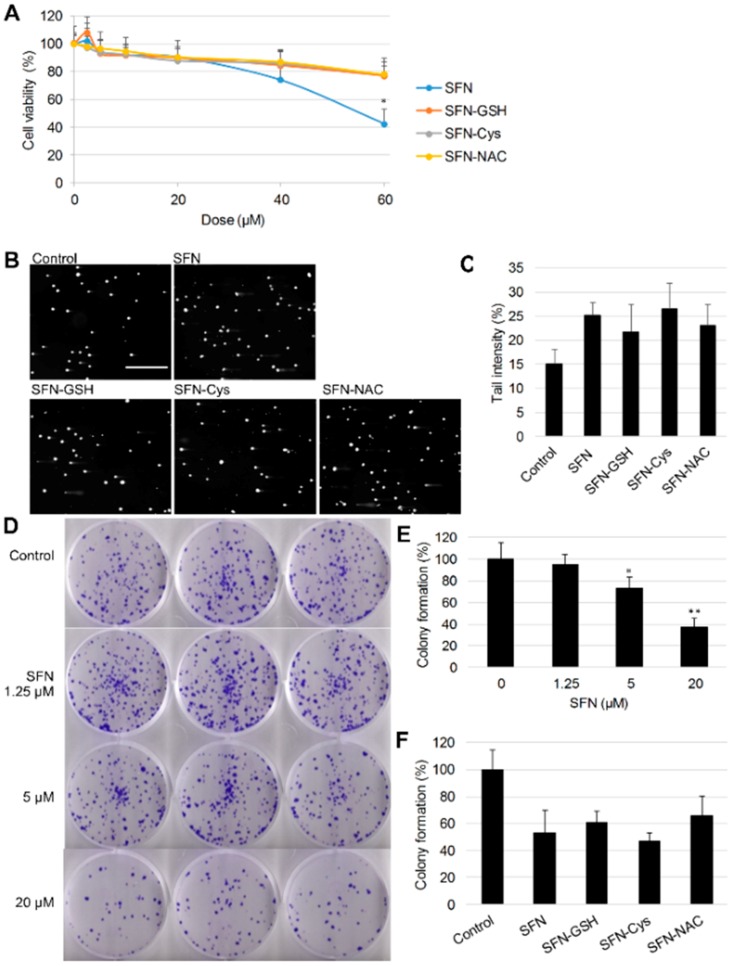
Effect of SFN and its metabolites on cell viability, genotoxicity and colony formation of HepG2 cells. (**A**) Cells were treated with different doses of SFN or its metabolites for 24 h, then cell viability was determined by the 3-(4,5-dimethylthiazol-2-yl)-2,5 diphenyl tetrazolium bromide (MTT) assay with dimethyl sulfoxide (DMSO) (0.1%) as control. Results represent the mean ± SD (*n* ≥ 5). Statistical significance within groups treated with the same dose, * *p* < 0.05. (**B**) Cells were treated with 20 µM SFN or metabolites with DMSO (0.1%) as control for 24 h, then levels of DNA strand breaks were determined by alkaline comet assay. Representative pictures from the comet assay. Scale bar = 500 µm. (**C**) Tail intensity was measured for at least 100 comets per sample. Data are presented as means ± SD. (**D**) Cells were treated with 1.25, 5 and 20 µM SFN with DMSO (0.1%) as control for 24 h, then seeded into 6-well plates for colony formation. After 14 days of incubation, formed colonies were stained for photograph and quantified (**E**). Statistical significance from the control, * *p* < 0.05, ** *p* < 0.01. (**F**) Cells were treated with 10 µM SFN or metabolites with DMSO (0.1%) as control, followed by colony formation and quantification. Results represent the mean ± SD (*n* = 3).

The cytotoxicity of SFN and its metabolites was determined by MTT assay. As shown in Figure 2A, all compounds tested induced cytotoxicity in a dose-dependent manner after 24 h treatment. At the highest concentrations tested, SFN showed stronger cytotoxicity than all three of its metabolites (*p* < 0.01), but there were no significant differences between the cytotoxicity of the metabolites. The genotoxicity of SFN and its metabolites were examined using the alkaline comet assay, using doses of 20 µM to avoid strong cytotoxicity. All compounds induced significant DNA damage compared to controls, while there were no significant differences between the genotoxicity of SFN and its metabolites (Figure 2B,C).

To further evaluate the effects of SFN and its metabolites on cancer cell growth, a colony formation assay was conducted. Fewer colonies formed following SFN treatment compared to controls (Figure 2D), and quantitative results indicated that SFN suppressed the formation of colonies in a dose-dependent manner (Figure 2E). A concentration of 10 µM was chosen to compare SFN with its metabolites (Figure 2F), and no significant difference was observed between the inhibitory effect of SFN and its metabolites on HepG2 colony formation.

### 3.2. Effect of SFN vs. Its Metabolites on Cancer Cell Migration

Given that the migration of cancer cells is an essential step for tumor metastasis and cell adhesion ability could help tumor cells colonize at new sites during metastasis [35], the effects of SFN and its metabolites on cell migration and adhesion were investigated.

**Figure 3:**
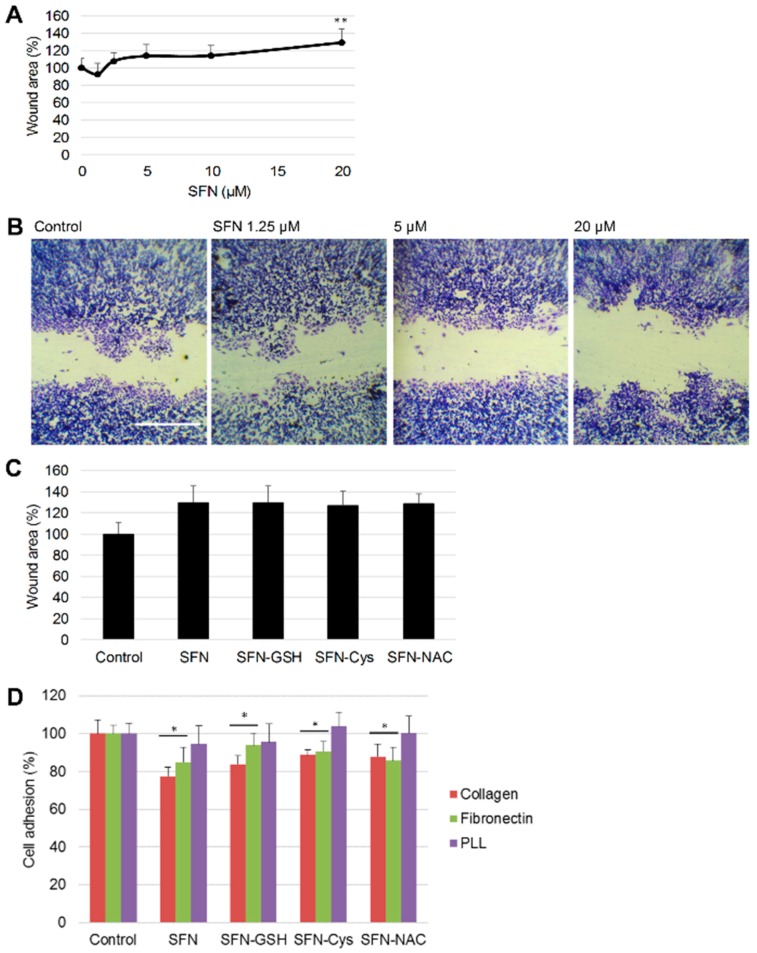
Effect of SFN and its metabolites on HepG2 cell migration and adhesion. (**A**) Cells were treated with 1.25 to 20 µM SFN for 48 h with DMSO (0.1%) as control, wound areas are presented as mean ± SD (*n* ≥ 5), ** *p* < 0.01 compared to control. (**B**) Representative phase contrast images from the wound assay. Scale bar = 1 mm. (**C**) Cells were treated with 20 µM SFN or metabolites for wound assay. Data are presented as means ± SD (*n* ≥ 5). (**D**) Cells were treated with 20 µM SFN or metabolites for 1.5 h with DMSO (0.1%) as control and was measured by adhesion assay. Data are presented as mean ± SD (*n* ≥ 6), * *p* < 0.05 compared to control. PLL: poly-l-lysine.

Wound assays were performed to measure HepG2 cell migration after 48 h under different doses of SFN treatment. Wound areas increased under SFN treatment (Figure 3A,B), and SFN inhibited cell migration of HepG2 cells in a dose-dependent manner. At 20 µM, SFN reduced cell migration to 70.8% compared to control. There was no significant difference between SFN and its metabolites in terms of their ability to inhibit cell migration (Figure 3C).

The effect of SFN and its metabolites on cell-extracellular matrix (ECM) interactions were measured by the adhesion assay. Plates were coated with two major kinds of ECM proteins–type I collagen and fibronectin–and PLL as a negative control for integrin-based cell adhesion. After a 1.5 h incubation, HepG2 cell adhesion ability under 20 µM SFN or its metabolites was quantified. Results showed that SFN suppressed adhesion of HepG2 cells on collagen and fibronectin but not PLL. Further comparison studies with 20 µM SFN or its metabolites in adhesion assays showed that the metabolites did not change the selectivity towards ECM and their inhibitory effects were not as strong as the effect of SFN (Figure 3D).

### 3.3. Effect of SFN vs. Its Metabolites on Angiogenesis

**Figure 4:**
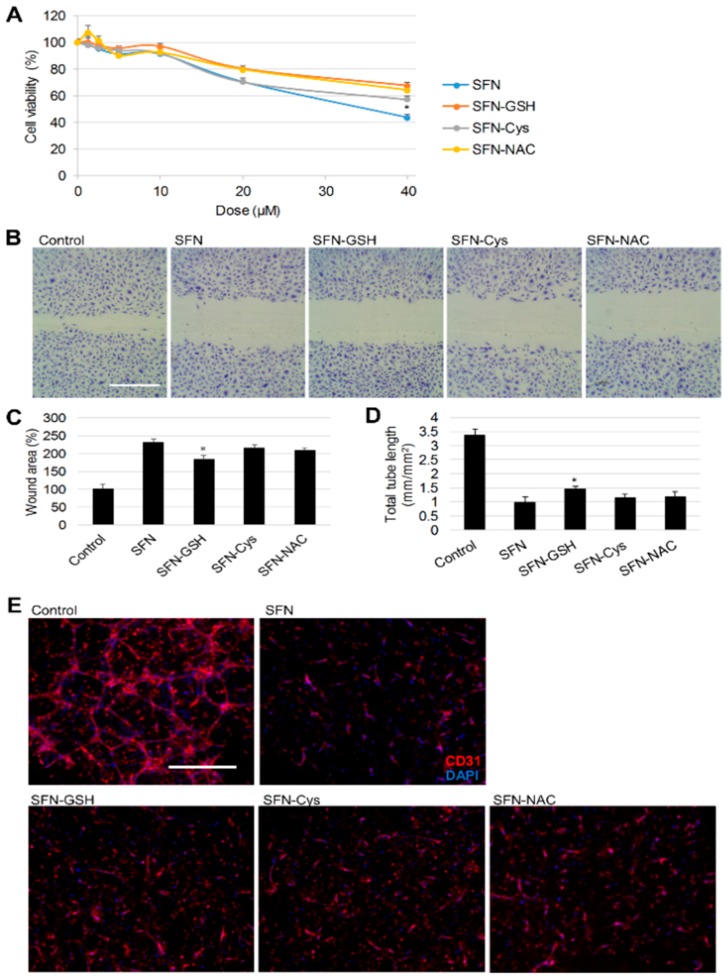
Effect of SFN and its metabolites on cell viability, migration and tube formation of human umbilical vein endothelial cells (HUVECs). (**A**) Cells were treated with different doses of SFN or its metabolites for 24 h, then cell viability was determined by MTT assay with DMSO (0.1%) as control. Results represent the mean ± SD (*n* ≥ 5). Statistical significance within groups treated with the same dose, * *p* < 0.05. (**B**) Representative phase contrast images from the wound assay under 20 µM SFN or metabolites treatment for 12 h with DMSO (0.1%) as control, scale bar = 1 mm. (**C**) Wound areas are presented as mean ± SD (*n* ≥ 5), * *p* < 0.05 compared to SFN treated group. (**D**) The total lengths of CD31 positive tubes formed in treated groups (10 µM SFN or its metabolites) with DMSO (0.1%) as control were measured and expressed as mean ± SD (*n* ≥ 3), * *p* < 0.05 compared to the SFN treated group. (**E**) Representative merged pictures from 3D co-cultures with CD31 endothelial cell marker (red) and DAPI nuclei staining (blue). Scale bar = 500 µm.

Angiogenesis, the formation of blood vessels from pre-existing vasculature, is required for both cancer progression and metastasis [36]. Previously, SFN (>5 µM) has been reported to inhibit HUVEC cell growth and migration, as well as HUVEC tube formation in a 3D pericyte co-culture model [37]. Here, the anti-angiogenic effects of SFN metabolites were examined and compared to that of SFN. Firstly, their effect on HUVEC cell viability was tested by MTT assay after 24 h treatment. A dose-dependent inhibition of cell viability was observed from these metabolites compared to the DMSO control, while SFN still showed the highest cytotoxicity (Figure 4A). Secondly, the effect of SFN metabolites on HUVEC migration was measured by the wound assay (Figure 4B,C). After 12 h, SFN-Cys and SFN-NAC exhibited inhibitory effects similar to that of SFN, indicated by a nearly 2-fold increase of wound area compared to controls, but SFN-GSH was less effective in inhibiting HUVEC cell migration (*p* < 0.01). Finally, the effect of SFN and its metabolites on tube formation was examined in the 3D co-culture HUVEC with pericytes model (Figure 4D,E). The average total tube length was 3.37 and 0.99 mm/mm^2^ in the control and SFN treated groups, respectively. At the same dose, there was no significant difference between this inhibitory effect using SFN, SFN-Cys or SFN-NAC; SFN-GSH however, showed modestly weaker inhibition (1.46 mm/mm^2^ total tube length). In summary, SFN was the strongest inhibitor of HUVEC cell viability, migration and tube formation, followed by SFN-Cys and SFN-NAC, but SFN-GSH showed the weakest inhibitory effect among all the metabolites.

### 3.4. Protective Effect of SFN vs. Its Metabolites against H_2_O_2_

**Figure 5:**
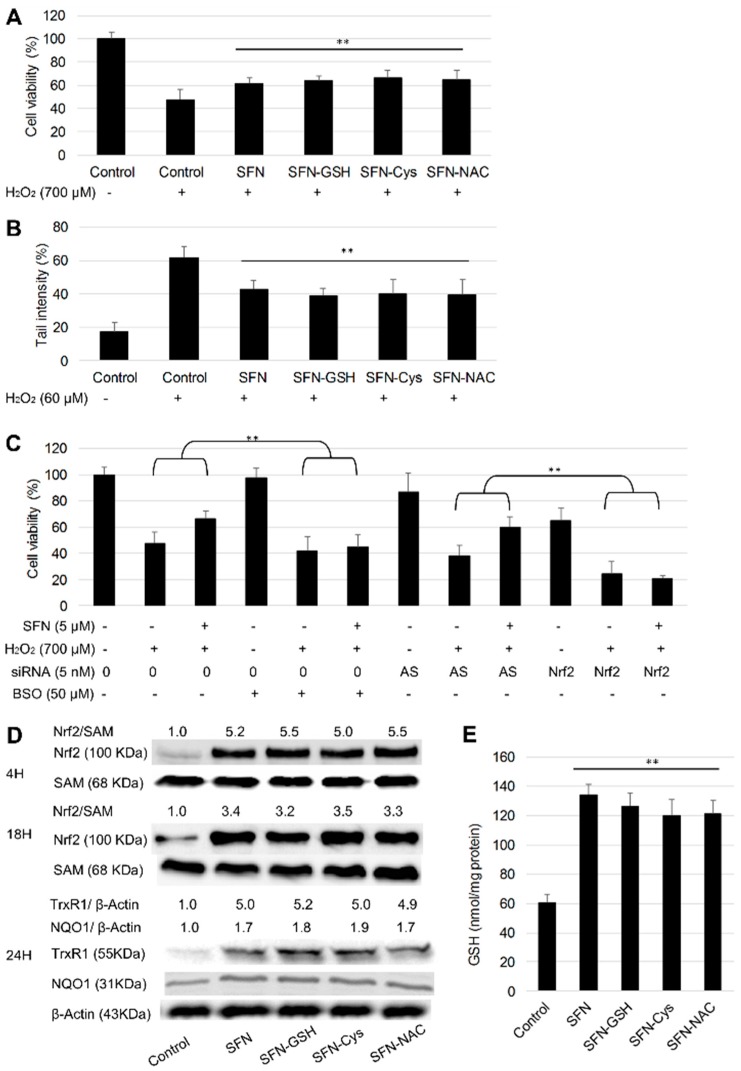
Protective effect of SFN and its metabolites. (**A**) HepG2 cells were pre-treated with 10 µM SFN or metabolites for 24 h and then incubated with H_2_O_2_ (+) or not (-) for another 24 h. Cell viability was measured by MTT assay, result represents the mean ± SD (*n* ≥ 5). ** *p* < 0.01 compared to H_2_O_2_ control. (**B**) Cells were pre-treated with 5 µM SFN or metabolites for 24 h and then incubated with H_2_O_2_ (+) or not (-) for another 30 min. DNA strand breaks were assessed by the alkaline comet assay. Tail intensities were measured for at least 100 comets per sample. ** *p* < 0.01 compared to H_2_O_2_ control. (**C**) Effect of nuclear factor E2-related factor 2 (Nrf2) knockdown and glutathione (GSH) inhibition on the protective effect of SFN against H_2_O_2_. Nrf2 was knocked down using siRNA as described in the Methods Section. Allstars (AS) was used as a negative control. Cells were incubated with 5 μM SFN or DMSO (0.1%) with or without 50 μM BSO for 24 h, then exposed to H_2_O_2_ for another 24 h. (+/-) indicates whether the treatments showed on the left were added. Cell viability was measured by MTT assay, results represent the mean ± SD (*n* ≥ 5), ** *p* < 0.01 between the indicated groups. (**D**) Effect of SFN and its metabolites on Nrf2 signaling activation. Cells were treated with 10 µM SFN or its metabolites for 4 or 18 h for nuclear protein extraction, or 24 h for whole protein extraction, DMSO (0.1%) was used as control. Nuclear Nrf2 was detected with SAM (Src-associated in mitosis 68 kDa, a RNA binding protein) as the loading control. Thioredoxin reductase 1 (TrxR1) and NAD(P)H quinone dehydrogenase 1 (NQO1) in whole cell lysates were detected with β-actin as the loading control. (**E**) Cells were treated with 10 µM SFN or its metabolites for 24 h with DMSO (0.1%) as control. The intracellular reduced GSH level was measured by HPLC, results were represented as the mean ± SD (*n* = 3), ** *p* < 0.01 compared to control.

HepG2 cells were pre-treated with 10 µM SFN or metabolites for 24 h, followed by 700 µM H_2_O_2_ treatment for another 24 h, and cell viability was then measured by MTT assay. Results showed that SFN pre-treatments reduced the cytotoxicity of H_2_O_2_, and all the metabolites exhibited a similar protective effect compared to SFN (Figure 5A). The protective effect from 5 µM SFN and its metabolites against H_2_O_2_ induced DNA strand breaks was also measured by the alkaline comet assay (Figure 5B). Cells were pre-treated with SFN or metabolites for 24 h followed by 60 µM H_2_O_2_ treatment for 30 min. The short incubation time of H_2_O_2_ treatment was designed to avoid the action of DNA repair mechanisms. Again, there was no significant difference between the protective effect of SFN and its metabolites.

Nrf2 is generally considered as the main transcription factor that regulates cellular defence mechanisms, especially against oxidative stress. It targets more than 200 genes, many of which provoke strong cytoprotective responses. Nrf2 controls the production, utilization and regeneration of glutathione (GSH), the most abundant antioxidant cofactor within cells [38]. Here, the role of Nrf2 and GSH in the protective effect of SFN was investigated. Cells were either transfected with siNrf2 (Allstar as negative control) and pre-treated with 5 µM SFN, or pre-treated with dl-Buthionine-sulfoximine (BSO), a specific inhibitor of γ-glutamylcysteine synthetase, along with SFN for 24 h, followed by H_2_O_2_ insult for another 24 h. Nrf2 knockdown enhanced the cytotoxicity of H_2_O_2_, i.e., cell viability was 47.6, 40.0 and 24.6% in the non-transfected, Allstar transfected and siNrf2 transfected cells, respectively (Figure 5C). SFN treatment (5 µM) decreased the cytotoxic effect of H_2_O_2_ in non-transfected and Allstar negative control cells. By contrast, the protective effect from SFN was abolished upon Nrf2 knockdown. Conversely, 50 µM BSO only reduced cell viability of HepG2 cells to 97.9%, while co-treatment with BSO and SFN showed no protective effect against H_2_O_2_. Therefore, it can be concluded that the Nrf2/GSH signaling pathway plays an essential role in the protective effect of SFN against H_2_O_2_.

Next, the activation of Nrf2/GSH signaling was examined under the treatment of SFN metabolites. SFN and its metabolites at 10 µM induced a clear increase of nuclear Nrf2 protein levels at 4 h and 18 h, and no significant difference was observed between SFN and its metabolites in the activation of Nrf2 translocation into the nucleus (Figure 5D). To determine whether the nuclear accumulation of Nrf2 by SFN and its metabolites resulted in the up-regulation of Nrf2 target genes, the expression of TrxR1 and NQO1 was measured after 24 h by Western blotting. Results showed significant increases of the target protein levels which suggests that the nuclear translocation of Nrf2 has a functional downstream effect. Again, the metabolites showed similar inductions of TrxR1 and NQO1 compared to SFN in both cell lines. Changes in intracellular reduced GSH levels at 24 h were measured using HPLC relative to controls. SFN and all three of its metabolites induced at least a 2-fold increase of GSH level in HepG2 cells, and there was no significant difference between SFN and its metabolites (Figure 5E).

## 4. Discussion

SFN can suppress cancer development through various molecular targets. It has been shown to induce DNA single [39] or double-strand [29] breaks in cancer cells that link to the production of reactive oxygen species. On the other hand, SFN could impair critical DNA repair proteins and cause cancer cell growth arrest, autophagy and apoptosis [40,41,42]. It also inhibits cell migration in various human cancer cell models, the mechanism behind which can be associated with the suppression of the Hedgehog pathway [43] and epidermal growth factor receptor (EGFR) down-regulation [44]. Additionally, SFN could inhibit tumor growth by disrupting endothelial cell functions [45,46,47]. Here, three major metabolites of SFN were compared with the parent SFN in terms of their potential to inhibit cancerous phenotypes of HepG2 cells. The effects of SFN and its metabolites on cell viability, migration and the tube formation of HUVECs were also studied for the first time. SFN and the metabolites were equally effective in inducing significant DNA damage, as well as inhibiting colony formation and cell migration (10 and 20 µM). Notably, SFN selectively inhibited HepG2 cell adhesion on collagen compared to adhesion on fibronectin and poly-l-lysine; and its metabolites showed the same selectivity. This indicates that SFN and its metabolites influenced collagen-mediated cell adhesion which could be associated with the integrins alpha 1,2,10,11 in a heterodimer with beta1 [48,49]. In HUVEC cells, SFN-Cys and SFN-NAC exhibited similar inhibitory effects to SFN on cell viability, migration and tube formation, but the effects of SFN-GSH were significantly weaker. One possible explanation is that the transient depletion of GSH under SFN treatment [34] was alleviated compared to that under SFN-GSH, which reduced the inhibitory effects in cell migration and tube formation, as HUVEC cells are sensitive to the toxicity of SFN and its metabolites. Further study of the role of GSH in the differences observed between the bioactivities of SFN and SFN-GSH on HUVECs is needed.

Promoting the cytoprotective Nrf2 pathway has become an attractive target for chemoprevention. Previous studies have demonstrated that SFN induced Nrf2 and TrxR1 expression in dose- and time-dependent manners in human hepatocytes, and HepG2 cells [50,51]. Here, no significant difference was observed between SFN and its metabolites in terms of their ability to activate Nrf2 translocation or to induce phase II enzymes TrxR1 and NQO1. Further investigation of their effects on intracellular GSH showed that SFN and its metabolites induced at least a 2-fold increase of GSH after 24 h. These data highlight the potential chemopreventive effects of SFN metabolites. Additionally, these metabolites showed a similar protective effect against H_2_O_2_-induced cell death and DNA damage in HepG2 cells as SFN. As the metabolites were less toxic when compared to SFN at high doses (>40 µM), while still preserving similar protective effect at lower doses (2.5–10 µM), they could be used to reduce the risk of possible side effects on sensitive tissues, in this case hepatotoxicity, in the pursuit of achieving chemopreventive effects.

The results of this study indicated that in HepG2, both Nrf2 and GSH substantially contributed to the preservation of cell integrity against H_2_O_2_ insult, i.e., the inhibition of Nrf2/GSH decreased the cytoprotective effect of SFN against H_2_O_2_ in HepG2 cells. The knockdown of Nrf2 increased the levels of cell death even further compared to GSH inhibition, indicating that more Nrf2 targets might be involved. Essentially, Nrf2 protects not only normal cells but also transforming/cancer cells. With increasing amounts of evidence suggesting that Nrf2 is upregulated in cancer cells or resistant strains [52,53], and contributes to the aggressive cancer phenotype [54], it becomes more important to rationalize the usage of Nrf2 activators, such as SFN and its metabolites. More rigorous dose-response comparisons of efficacy versus toxicity need to be performed with consideration of the differences between normal and cancer cells.

In summary, this study confirmed that at higher doses (>20 µM), three major metabolites of SFN (SFN-GSH, SFN-Cys and SFN-NAC) decreased cell viability, induced DNA strand breaks, and inhibited tumorigenicity of HepG2 cells. In addition, they impaired angiogenic processes such as EC proliferation, migration and tube formation. On the other hand, pre-treatments of SFN or its metabolites at lower doses (2.5–10 µM) protected against H_2_O_2_-induced cell damage in HepG2 cells. There were no significant differences between SFN and its metabolites in the activation of Nrf2 and downstream gene expression, as well as the induction of intracellular reduced GSH. These results indicate that high doses of SFN and its metabolites could contribute to the inhibition of the progression of premalignant lesions, while at low doses they could act as primary chemopreventive agents. This study provides appealing evidence that the principal metabolites of SFN retain the anticancer activity of the parent compound in HepG2 cells, and that it is necessary to study SFN metabolites in other cancer types using both in vitro and in vivo models.
